# Speech intelligibility in Parkinson's disease patients with zona incerta deep brain stimulation

**DOI:** 10.1002/brb3.394

**Published:** 2015-09-25

**Authors:** Linda Sandström, Patricia Hägglund, Louise Johansson, Patric Blomstedt, Fredrik Karlsson

**Affiliations:** ^1^ Division of Speech and Language PathologyDepartment of Clinical SciencesUmeå UniversityUmeåSweden; ^2^ Division of Clinical NeuroscienceDepartment of Pharmacology and Clinical NeuroscienceUmeå UniversityUmeåSweden

**Keywords:** Deep brain stimulation, Levodopa, Parkinson's disease, posterior subthalamic area, spontaneous speech intelligibility, zona incerta

## Abstract

**Objectives:**

To investigate the effects of l‐dopa (Levodopa) and cZi‐DBS (deep brain stimulation in caudal zona incerta) on spontaneous speech intelligibility in patients with PD (Parkinson's disease).

**Materials and Methods:**

Spontaneous utterances were extracted from anechoic recordings from 11 patients with PD preoperatively (off and on l‐dopa medication) and 6 and 12 months post bilateral cZi‐DBS operation (off and on stimulation, with simultaneous l‐dopa medication). Background noise with an amplitude corresponding to a clinical setting was added to the recordings. Intelligibility was assessed through a transcription task performed by 41 listeners in a randomized and blinded procedure.

**Results:**

A group‐level worsening in spontaneous speech intelligibility was observed on cZi stimulation compared to off 6 months postoperatively (8 adverse, 1 positive, 2 no change). Twelve months postoperatively, adverse effects of cZi‐DBS were not frequently observed (2 positive, 3 adverse, 6 no change). l‐dopa administered preoperatively as part of the evaluation for DBS operation provided the overall best treatment outcome (1 adverse, 4 positive, 6 no change).

**Conclusions:**

cZi‐DBS was shown to have smaller negative effects when evaluated from spontaneous speech compared to speech effects reported previously. The previously reported reduction in word‐level intelligibility 12 months postoperatively was not transferred to spontaneous speech for most patients. Reduced intelligibility due to cZi stimulation was much more prominent 6 months postoperatively than at 12 months.

## Introduction

Parkinson's disease (PD) may affect patients' clarity of speech in a way that is traditionally classified as hypokinetic dysarthria, affecting patients' speech motor control and phonatory functioning. Speech effects vary in severity, with 52% of patients showing mild‐to‐moderate impairment, and 22% having severe or profound speech impairment (Ho et al. 1998). It has been estimated that 45–50% show articulatory effects (Logemann et al. 1978), whereas 80–90% of patients may be affected when the estimated prevalence also include voice and effects related to respiratory functioning (Logemann et al. 1978; Hartelius and Svensson 1994). The resulting reduced speech intelligibility has been reported by patients as a prominent effect of the disease (Miller et al. 2007).

In the last two decades, DBS (deep brain stimulation) has become an established alternative method for the treatment of advanced PD. It is associated with minimal morbidity and is effective also for PD patients that have ceased to respond l‐dopa (Levodopa) treatment (Breit et al. 2004). DBS has generally been found to improve gross motor functioning as well as patients' quality of life (Daniels et al. 2011), but has also been shown a potential for causing a variety of speech‐related side effects in several neurological conditions (e.g., Blomstedt et al. 2011b; Karlsson et al. 2011, 2013, 2014; Lundgren et al. 2011; Fytagoridis et al. 2012, 2013). For patients with PD, traditional targets include the STN (subthalamic nucleus) (Benabid 2003) and the GPi (globus pallidus pars interna) (Rodriguez‐Oroz et al. 2005). For STN‐DBS, occurrences of reduction in speech quality have been reported as a result of stimulation (Krause et al. 2004; Rodriguez‐Oroz et al. 2005; Törnqvist et al. 2005; Fasano et al. 2010; Sidtis et al. 2012; Eklund et al. 2014). Consequently, adverse effects of STN‐DBS on intelligibility has also been observed (Rousseaux et al. 2004; Törnqvist et al. 2005; Klostermann et al. 2008; Tripoliti et al. 2008, 2011, 2014; Sidtis et al. 2012). l‐dopa has been shown to have beneficial effects on intelligibility in a majority of studies (Rousseaux et al. 2004; De Letter et al. 2007a,b); one study, however, showed a nonsignificant reduction due to l‐dopa (Plowman‐Prine et al. 2009). When combined with STN‐DBS, results have indicated a strengthening of adverse effects of STN‐DBS even further (Rousseaux et al. 2004). For GPi‐DBS, overall UPDRS evaluations have not indicated stimulation induced adverse speech effects to the same degree as STN‐DBS (Rodriguez‐Oroz et al. 2005), but has also not provided an as beneficial effect in terms of the desired reduction in l‐dopa daily dosage of patients (Anderson et al. 2005; Rodriguez‐Oroz et al. 2005). To the best of our knowledge, the effect of GPi‐DBS on speech intelligibility of PD patients has not been assessed in a blinded procedure. However, a recent blinded evaluation of patients with primary dystonia using a blinded transcription task performed by naive listeners indicated no amelioration of speech intelligibility due to GPi‐DBS (Risch et al. 2015).

More recently, the posterior subthalamic area has received attention as promising target for DBS in the treatment of motor symptoms of neurodegenerative disorders (Fytagoridis and Blomstedt 2010; Timmermann et al. 2011; Fytagoridis et al. 2012; Xie et al. 2012; García‐Gomar et al. 2013). Specifically, effects of DBS in the caudal part of the zona incerta (cZi) has been investigated for both essential tremor (Plaha et al. 2009; Blomstedt et al. 2011a; Xie et al. 2012) and PD (Kitagawa et al. 2005; Plaha et al. 2006, 2009). Although effective on overall motor symptoms, reports from our group indicate that stimulation of the cZi may introduce specific adverse effects on speech functions in patients with PD. A deterioration has been observed in patients 12 months postoperatively in both accuracy and articulatory rate in simple speech motor tasks (Karlsson et al. 2011) as well as an increase in misarticulation of speech sounds requiring either a full range of articulatory movements (Karlsson et al. 2014) or good articulatory control (Eklund et al. 2014). PD has been indicated to reduce the ability for short time regulation of phonation during speech (Weismer 1984; Bunton and Weismer 2002; Goberman and Blomgren 2008), which has been indicated to remain (Karlsson et al. 2012) or possibly be reduced further (Eklund et al. 2014) by cZi‐DBS (deep brain stimulation in caudal zona incerta). As a consequence of these speech production effects, an overall reduction in perceived speech quality has been observed (Eklund et al. 2014) which may affect intelligibility, that is, “the degree to which the speaker's intended message is transmitted to the listener by means of the acoustic speech signal without any contextual cues, such as linguistic cues or visual cues from nonverbal communication” (Yorkston et al. 1996). The one study available in which intelligibility was assessed, also performed by our group (Johansson et al. 2014), investigated the intelligibility of single words extracted from read speech produced 12 months postoperatively (Post‐op). The assessment was performed in a condition with an added low‐level background noise by 32 naive raters in a blinded and randomized procedure. The results showed a stronger presence of adverse effects than of positive treatment outcomes in the individual patients, and an overall negative effect on the group level (Johansson et al. 2014). The added background noise level was chosen to simulate that of a library setting and was introduced in order to make sure that the assessment of intelligibility was not performed under listening conditions that may artificially inflate the speech intelligibility estimate. The low‐level background noise was shown by Johansson et al. to decrease intelligibility overall, and was further suggested to interact with adverse articulatory effect of cZi‐DBS to produce substantial reductions in intelligibility for individual patients (Johansson et al. 2014).

Thus, our previous evaluation of speech intelligibility indicated primarily adverse effects of cZi on intelligibility and especially in listening conditions with background noise, a factor that has received increased recent attention in connection with clinical assessment of dysarthric speakers (Ryherd et al. 2012). The method used in our previous assessment of intelligibility effects of cZi‐DBS included blinded transcription of single words extracted from a read speech passage. Single word assessments have been used to evaluate intelligibility in clinical populations with reduced speech proficiency (e.g., De Letter et al. 2007a; Haley and Martin 2011; Falk et al. 2012; Kim and Nanney 2014). The high level of control over productions resulting from using read speech support direct comparisons of the same tokens produced by the same patient under off and on treatment conditions. However, it is possible that the use of single words in the evaluation may inflate the impact of articulatory effects of treatment on the results that would not be as prominent in longer speech samples. Therefore, while allowing for direct comparison of speech before and after treatment, and affording control over the effect of content, word‐level assessments may result in a too narrow estimate of intelligibility. Many previous reports have indicated strong differences in results between spontaneous speech and forms of controlled speech tasks for patients with neurological conditions (Kempler and Van Lancker 2002; Sidtis and Van Lancker Sidtis 2003; Van Lancker Sidtis et al. 2010; Van Lancker Sidtis et al. 2012). The primary aim of the present study was therefore to follow‐up on our previous assessment of speech intelligibility effects of cZi‐DBS, this time using longer speech samples extracted from spontaneous speech. The secondary aim was to investigate whether the effects of cZi‐DBS observed 12 postoperatively may be assumed to be indicative of what is observed earlier in the postoperative time course, in this case the 6 months postoperative session. The tertiary aim was to compare the effect of cZi‐DBS with the effect of l‐dopa for the same patients.

## Method

### Participants

Eleven patients with idiopathic PD (two female, nine male, aged 46–71 years) were included in this prospective nonrandomized study. An overview of the patients is presented in Table 1. Patients had been selected on clinical grounds for bilateral DBS surgery in the cZi based on the assessment of overall motor function; no consideration was taken with regard to speech status. Eight of the included patients (P1–8) were also included in the previous assessment of intelligibility effects of cZi‐DBS (Johansson et al. 2014).

**Table 1:** Summary of the 11 bilaterally operated patients. Information on medication and ordinary LED dose (Tomlinson et al. 2010) is indicated for the Pre‐op session and the 6 and 12 months follow‐up sessions for patients P1–7, 9–11, along with group means and standard deviations. For P8, who received treatment for which LED is not available (Tomlinson et al. 2010) is not included in the group‐level LED mean, and the actual dosage is indicated instead. Means and standard deviations for age and disease duration, preoperative Hoehn and Yahr staging scale (Goetz et al. 2004) and median unified Parkinson's disease rating scale (Fahn and Elton 1987) motor scores (UPDRS‐III, item 18) are further indicated for the entire group of patients

Patient	Sex	Age at surgery (years)	Disease duration (years)	Hoehn and Yahr stage	UPDRS‐III, Item 18, score (Pre‐op)	Medication Pre‐op	LED (mg) Pre‐op	Medication 6 m Post‐op	LED (mg) 6 m Post‐op	Medication (12 m Post‐op)	LED (mg) 12 m Post‐op
1	F	66.6	10	2.5	1	l‐Dopa, Entacapone, Pramipexole, Rasagiline	637	l‐Dopa, Entacapone, Pramipexole, Rasagiline	410	l‐Dopa, Entacapone, Pramipexole, Rasagiline	387
2	M	71.4	7	2.5	2	l‐Dopa, Pramipexole	300	l‐Dopa, Pramipexole	450	l‐Dopa, Pramipexole	600
3	M	62.5	5	2	2	l‐Dopa, Entacapone, Pramipexole	1048	l‐Dopa, Entacapone, Pramipexole	1397	l‐Dopa, Entacapone, Pramipexole	1397
4	M	49.0	4	1.5	1	l‐Dopa, Entacapone, Pramipexole	1997	l‐Dopa, Entacapone, Pramipexole	1298	l‐Dopa, Entacapone, Pramipexole	1248
5	M	50.4	6	2.5	1	l‐Dopa, Entacapone, Pramipexole	1464	l‐Dopa, Entacapone, Pramipexole	1098	l‐Dopa, Entacapone, Pramipexole	2245
6	F	62.5	5	2.5	0	l‐Dopa, Entacapone, Pramipexole	1050	l‐Dopa, Pramipexole	848	l‐Dopa, Pramipexole	950
7	M	51.5	4	2	2	l‐Dopa, Pramipexole, Selegiline	1227	l‐Dopa, Pramipexole	924	l‐Dopa, Pramipexole	924
8	M	66.2	10	2	1	—	0	—	0	Pargitan mite 2 mg 1 × 3	NA
9	M	52.9	7	2.5	0	l‐Dopa, Entacapone, Pramipexole	1315	l‐Dopa, Pramipexole	752	l‐Dopa	675
10	M	45.8	13	2	1	l‐Dopa, Entacapone, Pramipexole	1746	l‐Dopa, Entacapone, Pramipexole	2246	l‐Dopa, Entacapone	1222
11	M	52.2	7	2	1	l‐Dopa, Entacapone, Pramipexole, Rasagiline	1009	l‐Dopa, Entacapone, Pramipexole, Rasagiline	1209	l‐Dopa, Entacapone, Pramipexole	1231
Summary	2F/9M	57.3 ± 8.7	7.1 ± 2.8	2.2 ± 0.34	1 (0–2)		1197 ± 591		1063 ± 598		1088 ± 593

LED, l‐dopa equivalent.

Recordings of 11 age‐ and sex‐matched NC (normal control) speakers (two female, nine male, aged 45–71; mean age 57.3 ± 8.7) with normal hearing and no known speech disorder were further included to form a basis for evaluation of speech effects in the patient group. All participants gave their written informed consent after receiving information about the details of the study, and were all native speakers of Swedish.

### Study design

Patients were recorded at three time points, and under two treatment conditions for each time point. The presurgical recording (Pre‐op) was performed off medication (Med OFF) in the morning and on medication (Med ON) approximately 1.5 h later. In the preoperative Med ON recording, the patients received 1.5 times the patient's ordinary dose of LED (l‐dopa equivalents) in order to ensure that they were in an “on” state during the evaluation for the DBS procedure (Deuschl et al. 2002). Postsurgical recordings were performed 6 and 12 months after cZi‐DBS surgery with stimulation turned off (Stim OFF) and on (Stim ON), 60 min after stimulation was turned on and off, respectively. All postsurgical evaluations were performed with patients receiving simultaneous l‐dopa medication.

### Speech material

The speech material on which this study was based consisted of spontaneous speech produced during a recording in which the patients performed a range of speech tasks. The elicitation was performed as part of a natural communication and was not scripted, structured, or controlled in any way. Recordings were made in a sound‐treated booth, using a calibrated head mounted microphone (Sennheiser MKE 2 P‐C), with a 15‐cm mouth‐to‐microphone distance. The speech samples were recorded using a digital audio flash recorder (Marantz PMD 660) or a digital audio tape recorder (Panasonic SV 3800) at 48‐kHz sampling rate. A calibration tone (80 SPL dB, 1 kHz) was included at the beginning of each recording to serve as a reference in the determination of speaking amplitude.

The content of the utterances made by the patients in the recordings were written down in connection with the recording using the Praat software package (Boersma and Weenink 2001). The entire corpus of transcribed utterances included 2790 utterances. Based on the written transcripts of all transcribed utterances, sentences suitable for inclusion in the intelligibility testing were selected from the contents. Sentences with infrequently used words or with severely marked syntactical structure were removed. The selection of utterances did not involve listening to the sentence as part of the evaluation for inclusion. In total, 231 utterances, 2–8 words of length (5.04 ± 1.22), 3–12 syllables (7 ± 1.9) were extracted: three utterances per session (Pre‐op, 6 and 12 months Post‐op) and treatment (Med or Stim ON/OFF) for PD speaker and three utterances per NC speaker. Three utterances were missing due to lack of utterances fulfilling the set of criteria for inclusion (P2 6 months Post‐op Stim ON, P4 Pre‐op Med ON, and P5 12 months Post‐op Stim ON). All utterances were submitted to a calibration procedure, where their amplitudes when spoken were restored using the recorded calibration tone. In our previous study (Johansson et al. 2014), the amplitudes were normalized using the reference tone to capture differences in amplitudes when produced, but not restored to an actual playback amplitude corresponding to what would reach the ear of a listener in spoken conversation. The amplitude of each utterance was subsequently reduced by 6 dB, simulating a 1‐m talking distance. A background noise with amplitude of 44.9 dB was added to each utterance, simulating a more naturalistic talking environment. The amplitude of the background noise was calibrated against a reference recording made in a clinical setting. The background noise contained no discernible words.

### Listening procedure

Forty‐one listeners (18 men, 23 women; mean age of 33.1 ± 12.84 years) participated in the listening tests. The listeners were native Swedish speakers with normal hearing and no known memory deficits. None of the listeners had previous experience with speech analysis.

The stimuli were presented through headphones, using stimulus presentation software (Hillenbrand and Gayvert 2005). All listening tasks were performed on the same computer and with the same headphones at a fixed volume calibrated to the recorded calibration tone. The playback volume was set so that the amplitude of the original utterance relative to the 1‐m talking distance was restored. The listeners were instructed to use the computer keyboard to type (transcribe) what they heard. They were encouraged to write down everything that they could perceive, regardless if what they perceived were “real words” or not. Each listener performed the task in a single session with the possibility to pause whenever needed. The stimuli were presented in random order, and the listeners were blinded to speaker identity and stimulation condition.

### Data analysis

The listeners' transcriptions were scored individually by three of the authors. Syllables were used as a base unit in accordance with recently developed methodology (Lagerberg et al. 2015; Risch et al. 2015). The scoring system was, however, extended to address issues observed in the responses. In our implementation, a score of 1 indicated that the syllable was correctly transcribed, either orthographically or phonologically. A score of 0 indicated that the syllable was incorrect or absent from the transcription. A score of −1 indicated that the listener's transcription contained more syllables than the target utterance. Intelligibility score was calculated as the percentage of correctly transcribed syllables. Each patient's total intelligibility score was subsequently computed for each treatment condition (Pre‐op Med OFF/Med ON, 6 months Stim OFF/Stim ON, and 12 months Stim OFF/Stim ON). The intelligibility scores of NC speakers were computed in the same way and at the same time as the scoring of PD patients in order to afford an assessment of the performance of the transcription method used. The raters were blinded to the identity and condition of the speaker while scoring.

### Statistical analysis

The effect of treatment (OFF and ON l‐dopa Pre‐op and OFF and ON cZi‐DBS Post‐op) and amplitude of the recording on listeners' percentage of correct scores in Pre‐op recordings was investigated using a repeated measures (within patient) ANOVA. Post hoc comparisons were performed using Turkey honestly significant difference testing.

## Results

**Figure 1:**
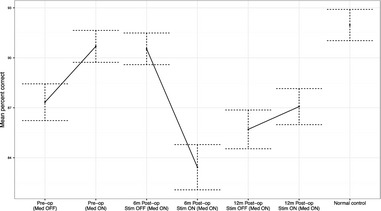
Plot of the mean portion of spontaneously produced utterances perceived correctly, in percent, across the entire group of patients and for normal control speakers. Mean portion across the investigated utterances as well as a 95% confidence interval for that mean estimate is indicated for productions made off and on medication in Pre‐op (preoperative) session, and off and on deep brain stimulation in caudal zona incerta stimulation 6 and 12 months in postoperative sessions.

The effect of cZi‐DBS was investigated 6 and 12 months Post‐op in a within patient ANOVA, with amplitude of the recording included as an additional covariate. The results showed a strong dependence on the location in time of the recording session (Fig. 1). In 6 months Post‐op recordings, results showed a significant decrease in intelligibility scores on cZi‐DBS compared to off (*F*_1,2663_ = 71.1, *P* < 0.001). At this point in the disease progression, mean percent correct at the group level decreased from 90.54% (SE = 0.485) off stimulation to 85.6% (SE = 0.624) on stimulation. Eight of the individual patients showed an effect of stimulation that agreed with the group‐level effect; one patient showed the opposite effect and two patients showed no effect (Table 2).

**Table 2:** Individual mean percent correct transcription scores. Transcription scores are given as means and SE are given for each patient, session (Pre‐op and 6 or 12 months Post‐op) and treatment condition (Med OFF or Med ON for Pre‐op stimuli and Stim OFF or Stim ON for Post‐op recordings). The best outcome and the result of the post hoc testing applied to investigate the off/on difference are further indicated for each patient. Patients 1–8 have been assessed previously in terms of effects of cZi stimulation on word‐level intelligibility 12 months Post‐op (Johansson et al. 2014)

	Pre‐op				6 months Post‐op				12 months Post‐op			
Patient	Med OFF	Med ON			Stim OFF	Stim ON			Stim OFF	Stim ON		
	Mean ± SE	Mean ± SE	Best outcome	Off/on comparison	Mean ± SE	Mean ± SE	Best outcome	Off/on comparison	Mean ± SE	Mean ± SE	Best outcome	Off/on comparison
P1	98.6 ± 0.89	89.4 ± 1.27	Med OFF	*P* < 0.001	83.1 ± 2.05	96.8 ± 0.77	Stim ON	*P* < 0.001	85.2 ± 1.71	82.2 ± 2.56		*P* = 0.32
P2	92.9 ± 1.20	79.0 ± 2.25	Med OFF	*P* < 0.001	95.3 ± 0.69	91.1 ± 1.52	Stim OFF	*P* = 0.01	93.8 ± 0.91	86.2 ± 1.26	Stim OFF	*P* < 0.001
P3	94.5 ± 2.92	80.7 ± 2.00	Med ON	*P* < 0.001	92.0 ± 1.82	47.0 ± 2.75	Stim OFF	*P* = 0.001	84.4 ± 1.73	75.5 ± 2.32	Stim OFF	*P* = 0.003
P4	80.7 ± 2.00	96.5 ± 1.72	Med ON	*P* < 0.001	90.4 ± 1.95	93.1 ± 1.13		*P* = 0.22	95.4 ± 1.03	93.6 ± 1.24		*P* = 0.26
P5	88.1 ± 1.62	93.0 ± 1.44	Med ON	*P* = 0.02	76.0 ± 2.08	83.8 ± 2.46	Stim OFF	*P* = 0.02	83.0 ± 2.51	81.1 ± 3.39		*P* = 0.65
P6	94.2 ± 1.38	97.1 ± 0.62	Med ON	*P* = 0.05	95.9 ± 0.59	94.8 ± 1.00		*P* = 0.36	91.8 ± 1.10	93.8 ± 1.19		*P* = 0.21
P7	87.2 ± 1.10	82.9 ± 2.6		*P* = 0.12	79.1 ± 1.67	68.0 ± 2.64	Stim OFF	*P* < 0.001	74.0 ± 1.92	92.0 ± 1.09	Stim ON	*P* < 0.001
P8	97.5 ± 0.56	97.6 ± 0.73		*P* = 0.96	99.1 ± 0.33	86.4 ± 2.37	Stim OFF	*P* < 0.001	98.5 ± 0.63	98.1 ± 0.64		*P* = 0.60
P9	81.0 ± 2.23	98.2 ± 0.59	Med ON	*P* < 0.001	97.1 ± 1.00	90.6 ± 1.13	Stim OFF	*P* < 0.001	90.4 ± 1.71	90.9 ± 1.18		*P* = 0.79
P10	90.3 ± 1.22	85.3 ± 1.09	Med OFF	*P* = 0.002	91.8 ± 1.64	81.3 ± 2.10	Stim OFF	*P* < 0.001	65.5 ± 2.85	88.2 ± 1.28	Stim ON	*P* < 0.001
P11	84.5 ± 2.00	87.8 ± 1.47		*P* = 0.17	97.2 ± 0.73	87.5 ± 1.41	Stim OFF	*P* < 0.001	80.8 ± 2.11	74.2 ± 1.96	Stim OFF	*P* = 0.02

SE, standard error.

At 12 months Post‐op, however, the results showed no significant group‐level effect of cZi‐DBS (*F*_1,2663_ = 2.8, *P* = 0.09, n.s.). Average scores were 85.7% (SE = 0.55) off stimulation compared to 87.1% (SE = 0.47) on stimulation. Six patients showed effects of stimulation that agreed with the group‐level effect (i.e., no significant effect of stimulation), two showed a positive treatment outcome of cZi‐DBS and three patients showed an adverse effect.

Compared to NC speakers, PD patients were found overall to be not significantly reduced in intelligibility off stimulation at 6 months (*F*_1,2824_ = 4.44, *P* = 0.44, n.s.). A reduced intelligibility in patients compared to NC speakers was, however, found on stimulation at 6 month (*F*_1,2783_ = 108.9, *P* < 0.001), as well as both off (*F*_1,2824_ = 71.8, *P* < 0.001) and on stimulation at 12 months (*F*_1,2783_ = 45.7, *P* < 0.001).

The effect of l‐dopa medication in Pre‐op recordings was investigated in a within patient ANOVA, with amplitude of the recording included as an additional covariate. The results showed a significantly higher percent correct transcriptions by the naive transcribers on l‐dopa medication compared to off (*F*_1,2622_ = 20.1, *P* < 0.001) (Fig. 1). On the group level, mean percent correct off medication was 87.4% compared to 93.0% (SE = 1.88) on medication. As indicated in Table 2, the group‐level pattern was observed in five of the individual patients; three patients showed an opposite effect and no difference between on and off medication states was established for three patients. PD patients were shown to be reduced in intelligibility compared to NC speakers in Med OFF (*F*_1,2824_ = 42.3, *P* < 0.001) but not in Med ON (Pre‐op) recordings (*F*_1,2742_ = 3.6, *P* = 0.06, n.s.) (Fig. 1).

## Discussion

The present investigation constitutes an expansion of the assessment of intelligibility of PD patients after cZi‐DBS reported previously (Johansson et al. 2014). The amplitude of the utterance at the time of production was restored, rescaled to mimic a conversation distance, and combined with a background noise at a level comparable to that of an assessment in a clinical setting. In contrast to the previous study, assessment of intelligibility was conducted based on full utterances produced in spontaneous speech. While the single word productions assessed in the previous report (Johansson et al. 2014) afforded strict control over the influence of the content of the produced speech on intelligibility, the use of spontaneous speech samples added a higher degree of confidence in the ecological validity (Brewer and Crano 2014) to the overall conclusion drawn. Inclusion of spontaneous speech samples, however, also introduced several additional sources of information to the listener, so that compensation for articulatory deficits is afforded to a greater extent. Inclusion of the 6 months Post‐op session (off and on stimulation) also added the ability to gauge effects of cZi‐DBS closely after the operation.

Compared to our previous report (Johansson et al. 2014), the spontaneous speech nature of the samples assessed here resulted in a reduced severity of found adverse effects of cZi stimulation. As such, the group effect of cZi‐DBS 12 months Post‐op showed greater benefit to intelligibility when assessed using spontaneous speech samples compared to the effect of STN‐DBS reported previously (Rousseaux et al. 2004; Törnqvist et al. 2005; Klostermann et al. 2008; Tripoliti et al. 2008, 2011, 2014; Sidtis et al. 2012). However, the presence of individual differences in treatment outcomes should also be considered. In our previous study, intelligibility was reduced on stimulation compared to off in eight of 10 patients when assessed in conditions that included a background noise. Two patients showed modest improvements (Johansson et al. 2014). In the present result, intelligibility was reduced on stimulation compared to off in only three of the patients. Six patients showed no significant effect of cZi‐DBS and two patients showed significant improvement. One of the two patients that showed improvement in the previous study (Johansson et al. 2014) was also shown here to improve by cZi stimulation (P7); the other patient showed no effect of stimulation when assessed on more complex speech. Thus, the agreement in overall interpretation of previously reported articulatory effects (Karlsson et al. 2011, 2012, 2014; Eklund et al. 2014) and reduced intelligibility at the word level (Johansson et al. 2014) is not transferred to spontaneously produced full utterances for all patients. Factors beyond speech articulation, such as intonation and rhythm, have been suggested to be at least moderately potent factors in determining intelligibility (Khan et al. 2013) to some speakers (Feenaughty et al. 2014). The present study additionally introduced an even higher level of control of speech amplitude in the assessments, a factor that has been highlighted recently in connection with assessments of intelligibility (Ryherd et al. 2012). Thus, linguistically richer samples and greater control over confounding factors were shown here to negate the adverse effects of cZi‐DBS that were observed previously in the 12 months Post‐op evaluation (Johansson et al. 2014).

While cZi stimulation was not shown here to have a substantial overall effect on speech intelligibility 12 months Post‐op, a stronger presence of adverse effects is seen earlier in the postoperative time course. In 6 months Post‐op recordings, adverse effects on speech intelligibility are more prominent, and is seen in eight of the 11 patients. One patient showed an improvement that was not present in the 12 months postoperative recording. The transient nature of both adverse and positive effects of cZi‐DBS on intelligibility 6 months Post‐op is consistent with microlesional effects (Rezai et al. 2006; Fytagoridis and Blomstedt 2010), possibly combined with the effects of disease progression. The need to treat assessments at 6 months follow‐up sessions with some caution regarding interpretation is highlighted by the results, as adverse effects of stimulation observed there may be more serious than what is observed only 6 months later. It is also possible that the “fine tuning” of the stimulation parameters and the balance between stimulation and medication has not been completely optimized at 6 months.

Of the effects investigated here, the best treatment outcome in terms of intelligibility was observed for l‐dopa administered as part of the evaluation for DBS operation. High levels of l‐dopa have not been previously assessed in terms of effects on intelligibility. On the group level, patients were shown to improve preoperatively on l‐dopa compared to off. On the level of individual patients, five of the 11 patients showed improved intelligibility on mediation compared to off preoperatively. Adverse effects were shown for three patients. The results presented here therefore lend further support to the beneficial effect of patient's ordinary l‐dopa dose on intelligibility found in a majority of previous studies (Rousseaux et al. 2004; De Letter et al. 2007a,b), even when given at an increased dose. For STN‐DBS, previous reports have indicated a particular adverse effect on speech intelligibility when combined with l‐dopa treatment (Rousseaux et al. 2004; Tripoliti et al. 2014). With Post‐op evaluations being performed on medication irrespective of cZi‐DBS treatment condition, the possibility of adverse or null effects of cZi‐DBS may in part be due to interaction between the two treatments. Separation of treatment effects and their interaction is, however, not afforded by the present research design and should be the target of further research.

## Conflict of Interest

L. Sandström, P. Hägglund, L. Johansson, and F. Karlsson have no financial disclosures. P. Blomstedt is a consultant for Medtronic and a shareholder in Mithridaticum AB.
